# A systematic review on the chemical constituents of the genus *Consolida* (Ranunculaceae) and their biological activities

**DOI:** 10.1039/d0ra06811j

**Published:** 2020-09-22

**Authors:** Tianpeng Yin, Le Cai, Zhongtao Ding

**Affiliations:** Zhuhai Key Laboratory of Fundamental and Applied Research in Traditional Chinese Medicine, Department of Bioengineering, Zhuhai Campus of Zunyi Medical University Zhuhai 519041 China; Functional Molecules Analysis and Biotransformation Key Laboratory of Universities in Yunnan Province, School of Chemical Science and Technology, Yunnan University Kunming 650091 China ztding@ynu.edu.cn caile@ynu.edu.cn

## Abstract

For centuries, species of the genus *Consolida* (Ranunculaceae) have been extensively utilized for their extremely high ornamental and medicinal values. Phytochemical investigations of *Consolida* species have revealed the presence of multiple active ingredients, including diterpenoid alkaloids, flavonoids, phenolic acids, phytosterols, fatty acids, and volatile constituents. These chemical constituents are of great research significance due to their novel structures and broad biological activities. This review addresses, for the first time, the chemical constituents of *Consolida* plants and the biological activities of these compounds to facilitate future research.

## Abstract 2

Multiple constituents from *Consolida* species showed great research significance for their novel structures and broad bioactivities.

## Graphical abstract

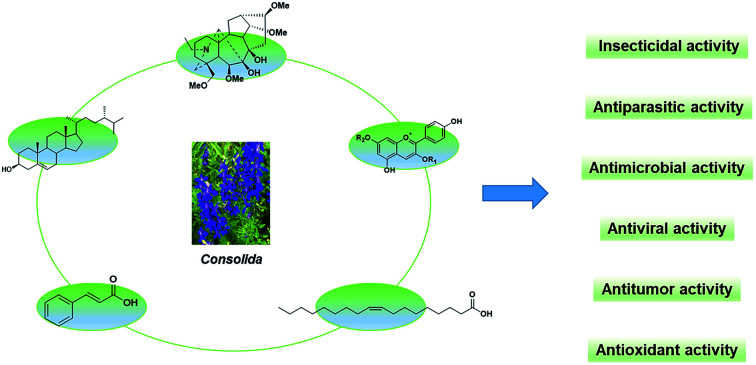

## 1. Introduction

**Fig. 1:**
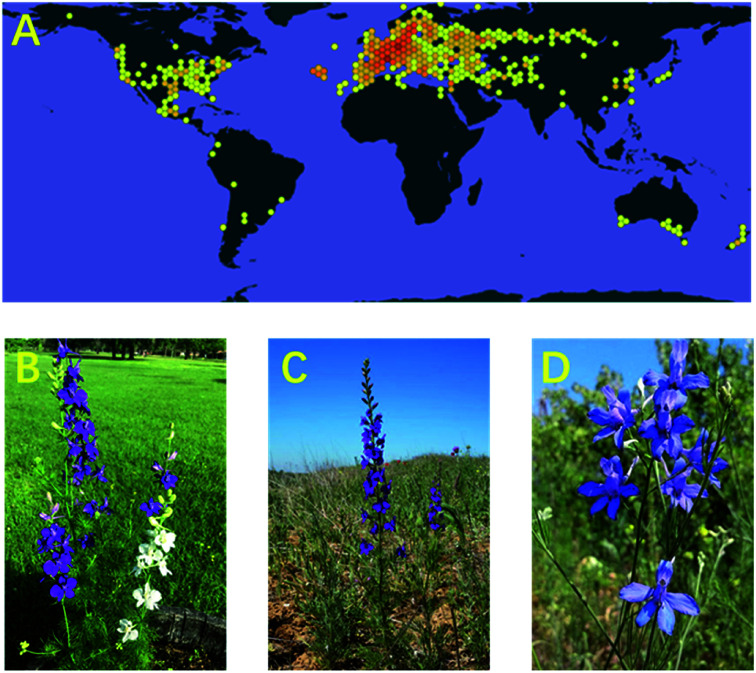
(A) The global distributions of plants from the genus *Consolida* (https://www.gbif.org/species/3033827); (B) *C. ambigua*, created by latormentanegra (https://www.inaturalist.org/observations/40694233); (C) *C. regalis*, created by Anastasiya Ishkova (https://www.inaturalist.org/observations/45139852); (D) *C. orientalis*, created by Sergei (https://www.inaturalist.org/observations/46362639).

The genus *Consolida*, a highly specialized genus of Ranunculaceae, is composed of approximately 50 species. *Consolida* plants are mainly distributed in drought regions in southern Europe, northern Africa, and western Asia, with a centre of diversity and speciation in Anatolia, as at least 29 *Consolida* species have been found in this region (Fig. 1A).^1,2^*Consolida* plants have adapted to the seasonal drought climate and often grow on dry stony slopes in steppes, semideserts, and even deserts. In addition, some of its representatives, such as *C. ambigua* (formerly known as *D. ajacis*) (Fig. 1B), have been widely cultivated in bonsai pots, gardens, and greenbelts around the world. Plants of the *Consolida* genus are morphologically very similar to those of *Delphinium* and are frequently mistaken. In fact, the *Consolida* genus has been treated as a phytogroup in the genus *Delphinium* for many years and was even given the same trivial name larkspur. However, in 1821, Gray raised *Consolida* to the species level, and now in most cases, *Consolida* is regarded as a different genus from the genus *Delphinium*.^3^ Generally, *Consolida* plants are annual herbals approximately 10–60 cm in height, possessing single petals and single follicles that distinguish them from *Delphinium* plants.^4,5^

Plants from the genus *Consolida* have received considerable interest due to their extremely high ornamental and medicinal values. *Consolida* plants feature showy purple petals, which have been widely cultivated for centuries not only as fresh and dried flowers but also as seasonal outdoor flowers. Some species of *Consolida*, such as *C. ambigua*, *C. regalis* (*D. consolida*) (Fig. 1C), and *C. orientalis* (Fig. 1D), have become some of the most famous and popular horticultural plants around the world, especially in Europe and America. In addition to ornamental plants, *Consolida* plants are also of great medicinal value. In Turkey, China, and some other countries and regions, especially the Mediterranean and western Asia, various *Consolida* species have been extensively employed as herbal medicines for hundreds of years to treat multiple kinds of diseases, such as traumatic injury, rheumatism, sciatica, enteritis, stomach ache, ringworm, scabies and other skin diseases.^6,7^ In addition, *Consolida* plants can also be used externally against body lice.^8^ Generally, the medicinal uses of *Consolida* plants are similar to plants from its highly related genus *Delphinium*, as they are similar in chemical composition.

The chemical constituents of *Consolida* plants have been investigated since the beginning of the 20th century. These earlier studies attempted to isolate and identify the alkaloidal and pigmental compositions of several widespread *Consolida* species, such as *C. ambigua* and *C. regalis*. In 1914, Keller and Voelker first reported the isolation of two diterpenoid alkaloids (DAs), ajacine and ajaconine, from the seeds of *C. ambigua*.^9^ The first anthocyanin, delphinin, was identified from the petals of *C. regalis* by Mieg in 1915.^10^ The DAs and flavonoids of *Consolida* plants have attracted considerable attention for a long period of time, and many phytochemical investigations have been devoted to them. In addition, a series of studies performed by using high-performance liquid chromatography (HPLC), gas chromatography (GC) or their combination with mass spectrometry (MS) techniques revealed that a large number of other chemical components, such as phenolic acids, phytosterols, fatty acids (FAs) and other volatile constituents, exist in *Consolida* plants. The constituents of *Consolida* plants have exhibited a high diversity of chemical structures and biological activities, and these constituents can serve as a potential medicinal resource for drug discovery.

Several already published review articles and monographs have involved the DAs from *Consolida*.^11–13^ However, to date, there has been no individual and systematic review of the chemical constituents in the genus *Consolida* in addition to their biological activities. Hence, this review has been prepared to summarize the structural features and biological activities of the chemical constituents in the genus *Consolida* for the first time. The aim of this review is to provide a complete overview on the existing knowledge of the chemical constituents and biological properties of plant species from *Consolida*, which will facilitate further research and exploitation of this genus.

## 2. Chemical constituents

To date, investigations on the chemical constituents of *Consolida* plants have led to the isolation and identification of approximately 143 distinct compounds, including 126 alkaloids and 17 flavonoids. In addition, phenolic acids, phytosterols, and FAs of several *Consolida* species have been investigated by using HPLC, GC, and MS methods. Herein, the studied chemical constituents of *Consolida* plants are summarized by category.

### 2.1. Alkaloids

In addition to the *Aconitum* and *Delphinium* genera, *Consolida* is another genus in the Ranunculaceae family that is well known for its characteristic DA components.^11,14^ DAs are unambiguously the most predominant and representative constituents of *Consolida* plants and have attracted much research interest since the beginning of the 20th century.^9^ However, studies on the DA composition of *Consolida* plants increased only in the 1980s due to the difficulty associated with the structural identification of DAs, which possess a fused polycyclic skeleton substituted with multiple oxygenated groups. To date, a total of 121 DAs (1–121) along with five other alkaloids (122–126) have been isolated from *Consolida* species. Table 1 lists the names, types, corresponding plant sources and references of alkaloids isolated and identified from *Consolida* species.

**Table 1:** Alkaloids from *Consolida* plants

Class and name (no.)	Type	Species	Ref.
**C**_**18**_ **-DAs**			
Hohenackeridine (1*)	I	*C. hohenackeri*	20
14-*O*-Demethyldelboxine (2*)	I	*C. orientalis*	21
14-Demethyltuguaconitine (3)	I	*C. orientalis*	26
Tuguaconitine (4)	I	*C. orientalis*	21
Lapaconidine (5)	II	*C. scleroclada*	27

**C**_**19**_ **-DAs**			
Pubescenine (6*)	III	*C. pubescens*, *C. oliveriana*, *C. orientalis*	8, 21 and 28
Hoheconsoline (7*)	III	*C. hohenackeri*	29
Consolinine (8*)	III	*C. hohenackeri*	29
Raveyine (8-*O*-methylcolumbianine, 9*)	III	*C. raveyi*, *C. oliveriana*	23 and 30
Regaline (10*)	III	*C. regalis*	31
Bicolorine (11)	III	*C. regalis*, *C. hohenackeri*	31 and 32
Senbusine B (12)	III	*C. anthoroidea*	33
Neoline (13)	III	*C. thirkeana*	34
14-*O*-Benzoylneoline (14)	III	*C. thirkeana*	34
Leucanthumsine C (15)	III	*C. thirkeana*	34
Neolinine (16)	III	*C. sulphurea*	34
Aconitine (17)	III	*C. scleroclada*	27
Delphisine (18)	III	*C. ambigua*	35
Ajadelphinine (19*)	III	*C. ambigua*, *C. orientalis*, *C. armeniaca*, *C. stenocarpa*	21, 35–37
Corepanine (20*)	IV	*C. regalis*	31
Hohenackerine (21*)	IV	*C. hohenackeri*	32
Tortumine (22*)	IV	*C. hohenackeri*	32
Delcorine (23)	IV	*C. regalis*, *C. hohenackeri*	31 and 32
Deoxydelcorine (24)	IV	*C. regalis*	31
Dehyrodelcorine (25)	IV	*C. regalis*, *C. hohenackeri*	31 and 32
Delcoridine (26)	IV	*C. regalis*	31
Didehydrodelsoline (27)	IV	*C. orientalis*	21
Deltaline (28)	IV	*C. ambigua*	38
Delpheline (29)	IV	*C. ambigua*	22
Ajacusine (30*)	IV	*C. ambigua*	39
Ajadine (31*)	IV	*C. ambigua*, *C. orientalis*	21 and 39
14-Deacetylajadine (32*)	IV	*C. ambigua*, *C. orientalis*	21 and 40
Ajadinine (33*)	IV	*C. ambigua*	24
19-Oxoanthranoyllycotonine (34*)	IV	*C. ambigua*	22
Ajacisine A (35*)	IV	*C. ambigua*	41
Ajacisine B (36*)	IV	*C. ambigua*	41
Ajacisine C (37*)	IV	*C. ambigua*	41
Ajacisine D (38*)	IV	*C. ambigua*	41
Ajacisine E (39*)	IV	*C. ambigua*	41
Delajacine (conambine, 40*)	IV	*C. ambigua*	38 and 42
Delajacirine (41*)	IV	*C. ambigua*	38
Delajadine (42*)	IV	*C. ambigua*	38
Ajanine (43*)	IV	*C. ambigua*	38
Ajacine (44)	IV	*C. ambigua*, *C. orientalis*	21 and 39
Anthranoyllycoctonine (45)	IV	*C. ambigua*, *C. oliveriana*	23 and 39
Delectine (46)	IV	*C. ambigua*	22
Isodelectine (47)	IV	*C. ambigua*	41
Methyllycaconitine (48)	IV	*C. thirkeana*, *C. axilliflora*, *C. ambigua*	34, 39 and 43
18-Hydroxy-14-*O*-methylgadesine (49*)	IV	*C. orientalis*, *C. oliveriana*	23 and 44
14-*O*-Acetyl-8-*O*-methylconsolarine (50)	IV	*C. orientalis*	21
18-Demethylpubescenine (51*)	IV	*C. orientalis*	26
Dehydrodeltatsine (52*)	IV	*C. orientalis*	45
14-*O*-Acetyltakaosamine (53*)	IV	*C. orientalis*	45
1-*O*-Demethyltricornine (54*)	IV	*C. orientalis*	21
14-*O*-Benzoyltakaosamine (55*)	IV	*C. orientalis*	21
1-*O*,19-Didehydrotakaosamine (56*)	IV	*C. orientalis*	21
8-*O*-Methylconsolarine (14-deacetyl-18-demethylpubescenine, 57*)	IV	*C. orientalis*	21 and 46
14-*O*-Deacetylpubescenine (58*)	IV	*C. orientalis*, *C. oliveriana*	21 and 23
18-*O*-Benzoyl-14-*O*-deacetyl-18-*O*-demethylpubescenine (59*)	IV	*C. orientalis*	21
18-Methoxygadesine (60*)	IV	*C. orientalis*, *C. ambigua*	35 and 47
Consolidine (61*)	IV	*C. oliveriana*	8
Olivimine (62*)	IV	*C. oliveriana*	23
Olividine (63*)	IV	*C. oliveriana*	23
1-Demethylwinkleridine (64*)	IV	*C. hohenackeri*, *C. anthoroidea*	20 and 33
18-Demethyl-14-deacetylpubescenine (65*)	IV	*C. hohenackeri*	20
14,18-Di-benzoyldelcosine (66*)	IV	*C. rugulosa*	48
14-Acetyl-18-benzoyldelcosine (67*)	IV	*C. rugulosa*	48
Ambiguine (68*)	IV	*C. ambigua*	49
14-Acetylbrowniine (69*)	IV	*C. ambigua*	39
Ajadelphine (70*)	IV	*C. ambigua*	35
19-Oxodelphatine (71*)	IV	*C. ambigua*, *C. oliveriana*	22 and 23
Paniculatine (72*)	IV	*C. regalis*	31
Paniculine (73*)	IV	*C. regalis*	31
Consolarine (74*)	IV	*C. armeniaca*	36
Gigactonine (75)	IV	*C. orientalis*, *C. ambigua*, *C. sulphurea*, *C. regalis*, *C. oliveriana*	8, 26, 34, 35 and 50
Delcosine (76)	IV	*C. orientalis*, *C. scleroclada*, *C. oliveriana*, *C. regalis*, *C. ambigua*	23, 26, 27, 39 and 50
Delbonine (77)	IV	*C. orientalis*	45
Deltatsine (78)	IV	*C. orientalis*, *C. ambigua*,	22 and 45
Delsoline (79)	IV	*C. oliveriana*, *C. orientalis*, *C. regalis*, *C. ambigua*	8, 39, 47, 48 and 50
Lycoctonine (80)	IV	*C. oliveriana*, *C. axilliflora*, *C. armeniaca*, *C. orientalis*, *C. ambigua*, *C. hohenackeri*, *C. regalis*	21, 23, 32, 36, 39, 43 and 50
Takaosamine (81)	IV	*C. orientalis*, *C. oliveriana*, *C. regalis*, *C. ambigua*, *C. axilliflora*	22, 23, 26, 43 and 50
Delphatine (82)	IV	*C. olopetala*, *C. oliveriana*, *C. ambigua*	6, 23 and 39
Delcaroline (83)	IV	*C. olopetala*	6
Browniine (84)	IV	*C. olopetala*, *C. oliveriana*, *C. sulphurea*, *C. ambigua*, *C. orientalis*	6, 21, 23, 34 and 39
14-Deacetylnudicaulidine (85)	IV	*C. sulphurea*	34
14-Benzoyldelcosine (86)	IV	*C. rugulosa*	48
14-Acetyldelcosine (87)	IV	*C. rugulosa*, *C. ambigua*, *C. orientalis*	21, 39 and 48
Potanine (88)	IV	*C. orientalis*	21
14-Deacetylambiguine (89)	IV	*C. ambigua*	22
Delectinine (90)	IV	*C. hohenackeri*, *C. axilliflora*	32 and 43
14-*O*-Acetyldelectinine (91)	IV	*C. orientalis*	21

**C**_**20**_ **-DAs**			
Azitine (92*)	V	*C. hellespontica*, *C. raveyi*	25
Chellespontine (93*)	V	*C. hellespontica*, *C. raveyi*	25 and 30
Consorientaline (94*)	V	*C. orientalis*	51
Dihydroajaconine (95*)	V	*C. ambigua*, *C. orientalis*, *C. oliveriana*	23, 46 and 49
Spiratine A (96)	V	*C. anthoroidea*	33
Atisine (97)	V	*C. regalis*, *C. anthoroidea*	33 and 50
Isoatisine (98)	V	*C. raveyi*	30
Ajaconine (99)	V	*C. anthoroidea*, *C. oliveriana*, *C. hohenackeri*, *C. ambigua*, *C. raveyi*, *C. axilliflora*	8, 30, 32, 33, 39 and 43
11,13-*O*-Diacetyl-9-deoxyglanduline (100*)	VI	*C. glandulosa*	52
13-*O*-Acetyl-9-deoxyglanduline (101*)	VI	*C. glandulosa*	52
14-*O*-Acetyl-9-deoxyglanduline (102*)	VI	*C. glandulosa*	52
13-*O*-Acetyl-glanduline (103*)	VI	*C. glandulosa*	52
Glanduline (104*)	VI	*C. glandulosa*	52
9-Deoxyglanduline (105*)	VI	*C. glandulosa*	53
Glandulosine (106*)	VI	*C. glandulosa*	53
11,13-*O*-Diacetylglanduline (107*)	VI	*C. glandulosa*	53
9-*O*-Acetylglanduline (108*)	VI	*C. glandulosa*	53
7α-Hydroxycossonidine (109*)	VI	*C. oliveriana*	23
13-*O*-Acetylvakhmatine (110*)	VI	*C. ambigua*	54
Vakhmatine (111)	VI	*C. ambigua*	54
Hetisine (112)	VI	*C. olopetala*, *C. anthoroidea*, *C. stenocarpa*, *C. axilliflora*	6, 33, 37 and 43
13-*O*-Acetylhetisine (113)	VI	*C. anthoroidea*	33
Septentriosine (114)	VI	*C. anthoroidea*	33
Hetisinone (115)	VI	*C. regalis*, *C. stenocarpa*	37 and 50
Leptanine (116*)	VI	*C. leptocarpum*	19
Stenocarpine (117*)	VII	*C. stenocarpa*	55
Willipelletierine (118*)	VII	*C. scleroclada*	27
Ajabicine (119*)	IX	*C. ambigua*	18
Dehydronapelline (120)	X	*C. olopetala*	6
12-Epidehydronapelline (121)	X	*C. olopetala*	6

**Other alkaloids**			
β-Carboline (122)		*C. ambigua*	41
Methyl-*N*-(3-carboxy-3-methylpropanoyl)anthranilate (123)		*C. ambigua*	41
2,4-Dihydroxy-1,4-benzoxazine-3-one 2-*O*-glucoside (124)		*C. ambigua*	56
2,4-Dihydroxy-1,4-benzoxazine-3-one (125)		*C. ambigua*	56
Benzoxazolinone (126)		*C. ambigua*	56

**Fig. 2:**
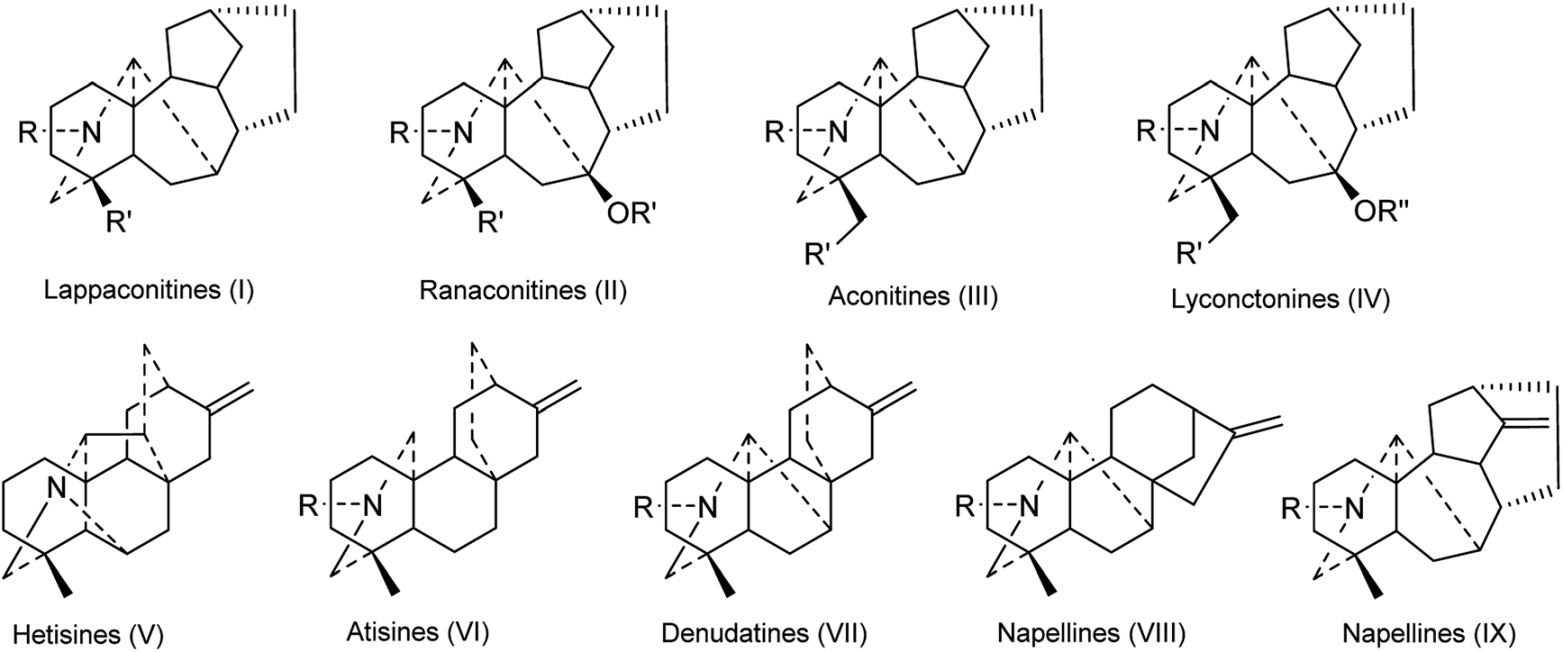
The subtypes of DAs from *Consolida* plants.

DAs are usually classified as C_18_-, C_19_-, C_20_- or bis-types, which can be further divided into several to dozens of subtypes.^15,16^ The DAs found in *Consolida* plants include 5 C_18_-DAs (1–5), 87 C_19_-DAs (5–91), and 29 C_20_-DAs (92–121). These alkaloids cover 9 subtypes of DAs, including the ranaconitine (I) and lappaconitine subtypes (II) of C_18_-DAs, the aconitine (III) and lycaconitine subtypes (IV) of C_19_-DAs, and the hetisine (V), atisine (VI), denudatine (VII), napelline (VIII), and other subtypes (IX) of C_20_-DAs (Fig. 2). In view of the chemical diversity, the lycaconitine-type C_19_-DAs contains the largest number of DAs in *Consolida* plants with 73 members, and they account for the largest proportion of isolated alkaloids (58%). The next largest subtypes are the hetisine-type C_20_-DAs with 17 members (13%) and the aconitine-type C_19_-DAs with 12 members (9%). Clearly, the lycaconitine-type C_19_-DAs are the most characteristic DA components of the genus *Consolida*, which is similar to its highly related genus *Delphinium*. In contrast, the large number of aconitine-type C_19_-DAs distinguishes *Consolida* from the genus *Aconitum*.^17^

**Fig. 3:**
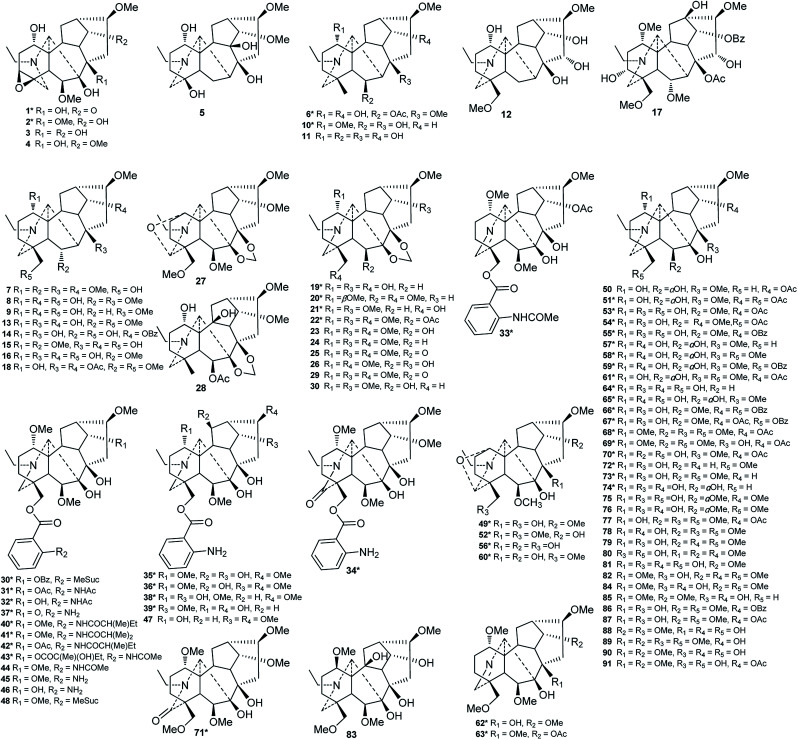
Alkaloids 1–91 from *Consolida* plants.

**Fig. 4:**
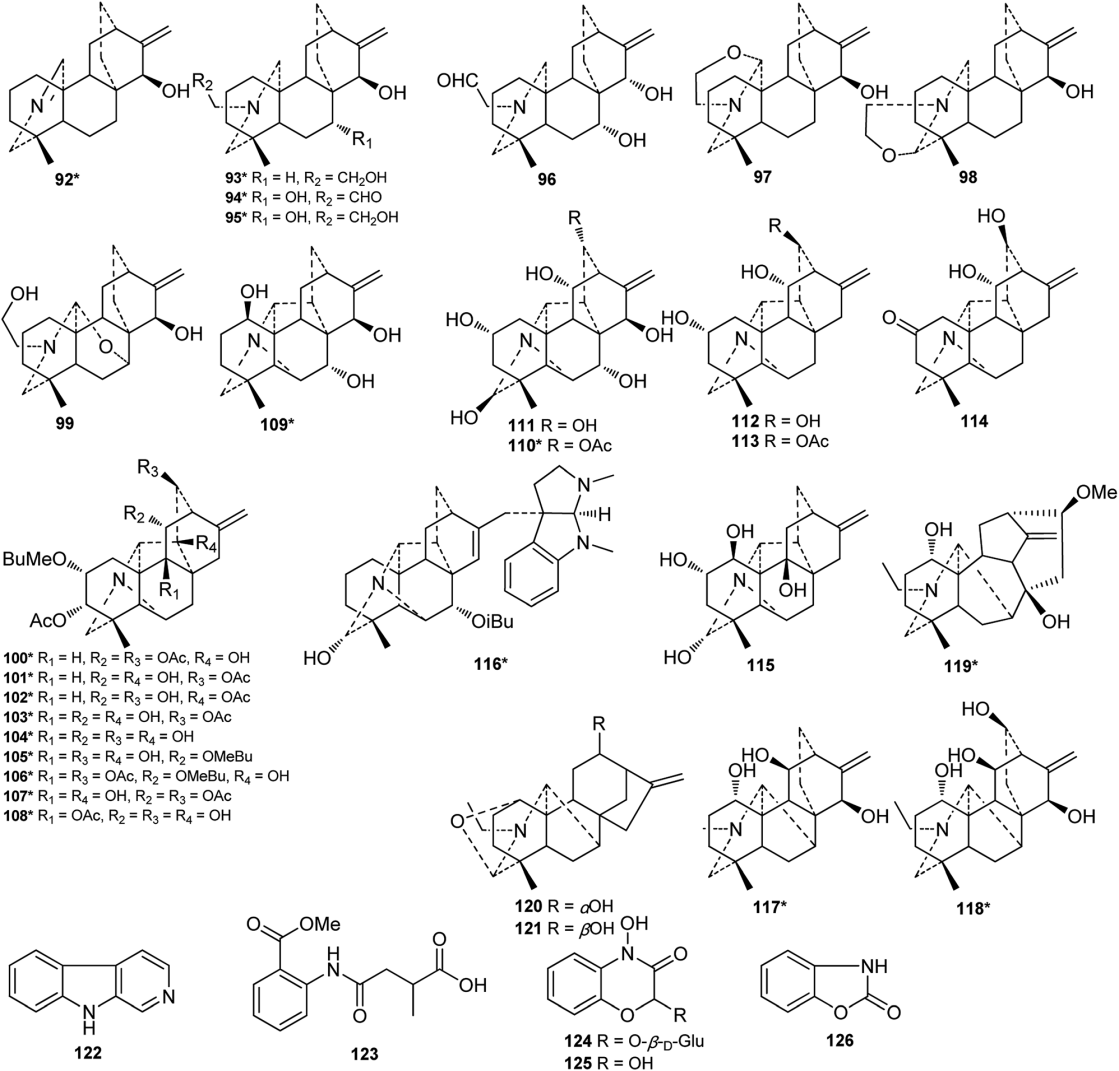
Alkaloids 92–126 from *Consolida* plants.

Of the 122 DAs presented in *Consolida* plants (Fig. 3 and 4), 69 were isolated as new compounds (labeled with *). Among them, several of the new alkaloids possess novel DA skeletons. Ajabicine (119) from *C. ambigua* belongs to the infrequent actaline-type C_20_-DAs bearing a rare C-14 exocyclic olefin methylene group, which may be produced biogenetically by a Wagner–Meerwein rearrangement of a denudatine-type DA.^12,18^ Leptanine (116) from *C. leptocarpum* (*D. leptocarpum*) is a dimeric alkaloid consisting of a hetisine-type C_20_-DA part and an indolinopyrrole fragment. The indolinopyrrole fragment is bound to the hetisine-type C_20_-DA part through an α-directed (relative to the indoline core) C-17–C-3 bond according to an X-ray crystal structure analysis.^19^ In addition, several of the new alkaloids possess at least one uncommon substituent. For example, new C_18_-DAs 1 and 2 possess an uncommon 3,4-epoxide unit,^20,21^ and new alkaloids 35 and 71 have an N–C_(19)_=O lactam group.^22,23^ New alkaloids 33, 62 and 63 possess an imine group at C-19,^23,24^ while alkaloid 92 has an imine group at C-17, a rare substituent position.^25^ The other new alkaloids mainly vary in the variety, quantity, position, and orientation of oxygenated substituents. The common oxygenated substituents found in DAs from *Consolida* plants include hydroxyl (OH), carbonyl (=O), methoxyl (OMe), methylenedioxy (OCH_2_O) groups and various ester groups, such as acetyl (Ac), 2-methylbutyryl (MeBu), benzoyl (Bz), and anthranoyl groups.

### 2.2. Flavonoids

Flavonoids, which are composed of C_6_–C_3_–C_6_ structural units biosynthesized from phenylalanine, are one of the most widespread types of natural products in the plant kingdom.^61,62^ The reported studies have revealed that a certain amount of flavonoids, including anthocyanin and flavanol glycosides, exist in *Consolida* plants, especially in their aerial parts.^63^

**Fig. 5:**
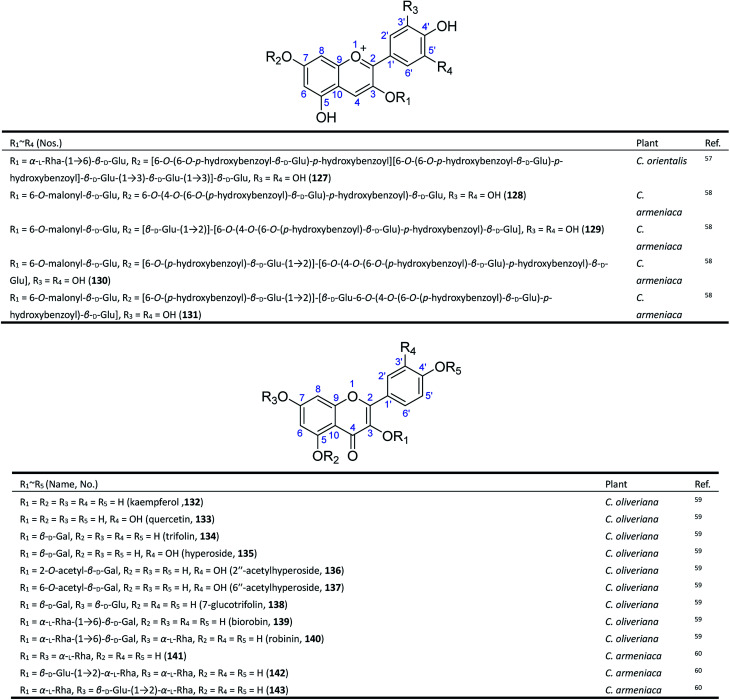
Flavonoids from *Consolida* plants.

Anthocyanins are the major pigments of *Consolida* flowers, which are of interest to the food industry because of their antioxidant power, attractive colour, and stability in highly acidic foods.^64,65^ As early as 1915, Mieg isolated the first anthocyanin delphinin from the purple petals of *C. regalis* (*D. consolida*) and proposed its structure to be di-(*p*-hydroxybenzoyl)delphin,^10^ but the existence of a *p*-hydroxybenzoyl group was doubted by Harborne in 1964.^66^ Finally, in 1975, Asen revised its structure as delphinidin 3-di-(*p*-hydroxybenzoyl)-glucosylglucoside.^67^ The reported discrepancies of the major anthocyanins found in *C. regalis* flowers may be attributed to the use of different plant materials, since there are a number of *C. regalis* varieties that have been cultivated all over the world. It should be noted that these early studies did not establish the location of substitutes and the linkage of glucoses in the molecules of anthocyanins until 1985. Sulyok and Balint yielded an anthocyanin from *C. orientalis* and identified its structure as delphinidin-3-rutinoside-5-glucoside (127) (Fig. 5).^57^ More recently, in 1995, four new acylated delphinidin 3,7-glycosides (128–131) were isolated from the blue-violet flowers of *C. armeniaca* as major anthocyanin pigments.^58^

The flavanol glycosides in *Consolida* have also drawn attention from scientists. Twelve known flavonol glycosides (132–143) have been isolated from two *Consolida* species, *C. oliveriana* and *C. armeniaca*.^59,60,68^ These flavonols only possess common structures but have attracted considerable interest because of their extensive pharmacological activities, including antitumor, antitrypanosomatid, and antioxidant activities.

### 2.3. Phenolic acids

**Fig. 6:**
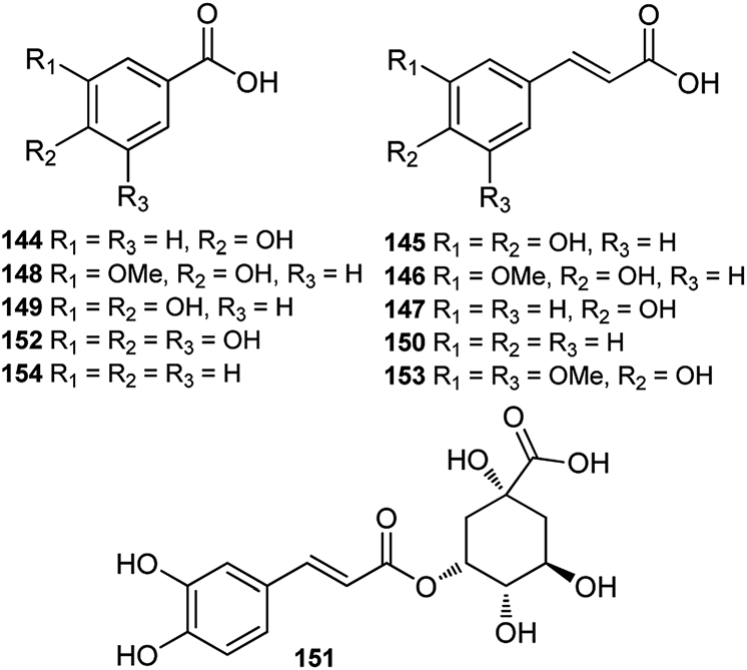
Phenolic acids from *Consolida* plants.

Until now, only a few studies on the phenolic acids of *Consolida* plants have been reported, and these studies were performed using HPLC or HPLC-MS techniques. A series of phenolic acids, mainly common phenylpropionic and benzoic acids, have been detected in the flowers of *Consolida* species (Fig. 6). For example, *p*-hydroxybenzoic (144), caffeic (145), ferulic (146) and *p*-coumaric (147) acids have been detected as the main phenolic compounds in *C. armeniaca* flowers,^69^ and protocatechuic (149), vanillic (148), cinnamic (150), chlorogenic (151), gallic (152), sinapic (153), and benzoic acids(154), in addition to acids 144–147, were identified in *C. orientalis* flowers.^70,71^

### 2.4. Phytosterols

**Fig. 7:**
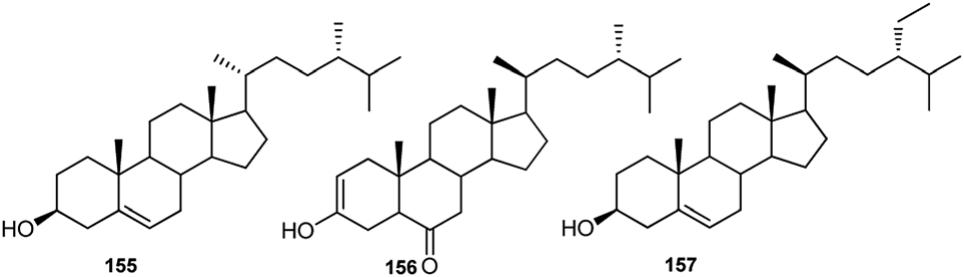
Main phytosterols in *Consolida* species.

Although phytosterols are widely distributed in higher plants, little attention has been paid to *Consolida* phytosterols. To the best of our knowledge, the only investigation on *Consolida* phytosterols was performed by Waller *et al.* in 1981.^72^ In this study, 16 phytosterols were identified and quantified from the whole *C. ambigua* plant using GC-MS, and the study revealed that the major sterols in the *C. ambigua* plants were β-sitosterol (155), campesterol (156) and stigmasterol (157) (Fig. 7).

### 2.5. Fatty acids and essential oils

**Fig. 8:**
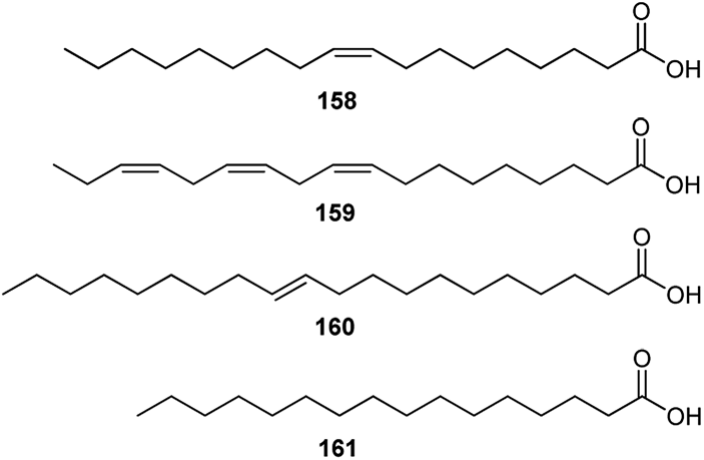
Main FAs in *Consolida* species.

Several studies have revealed that the seeds of *Consolida* species are a rich source of FAs. FAs are the major constituents of the oils from *Consolida* plants; for example, FA components are 87.16% of the seed oils of *C. regalis*.^73^ It has also been found that oleic acid (158), with a carbon chain length (CCL) of 18 : 1, is the most dominant FA in all studied *Consolida* plants (more than 50% of the total FAs), namely, *C. regalis*, *C. orientalis*, *C. armeniaca*, *C. glandulosa* and *C. hohenackeri* (Fig. 8).^73–76^*Consolida* plants also contain certain amounts of linoleic (159), eicosenoic (160), and palmitic (161) acids, whereas other FAs are almost negligible.

To date, only one species of *Consolida*, namely, *C. regalis*, has been investigated for its volatile constituents by using GC-MS,^73^ and a total of 66 compounds have been identified, representing 99.86% of the total content (Table 2). The analyses showed that the major constituents of the oils from *C. regalis* seeds were FAs (87.16%). In addition to the FAs, the carbonyl compounds (total content 8.57%), heptadecenal (3.58%), heptadecadienal (3.24%), and esters (total content 2.37%), particularly methyl octadecenoate (1.06%), were the main volatile constituents.

**Table 2:** Chemical constituents of oils from seeds of *C. regalis*

Compounds and class	CAS no.	Molecular formula	Relative content
**Hydrocarbons**			
2,6-Dimethyldecane	13150-81-7	C_12_H_26_	0.04
Undecane	1120-21-4	C_11_H_24_	0.03
Dodecane	112-40-3	C_12_H_26_	0.05
Tridecane	629-50-5	C_13_H_28_	0.07
Tetradecane	629-59-4	C_14_H_30_	0.04
Pentadecane	629-62-9	C_15_H_32_	0.02
Hexadecatriene	25167-60-6	C_16_H_28_	0.02
Hexadecane	544-76-3	C_16_H_34_	0.03
Heptadecadiene, isomer I	58045-14-0	C_17_H_32_	0.06
Heptadecadiene, isomer II	81265-03-4	C_17_H_32_	0.02
Heptadecane	629-78-7	C_17_H_36_	0.02
Octadecane	593-45-3	C_18_H_38_	0.03
Nonadecane	629-92-5	C_19_H_40_	0.02

**Carbonylic compounds**			
Nonan-2-one	30642-09-2	C_9_H_18_O	0.02
Nonanal	124-19-6	C_9_H_18_O	0.07
Non-2-enal	2463-53-8	C_9_H_16_O	0.02
Decan-2-one	693-54-9	C_10_H_20_O	0.02
Decanal	112-31-2	C_10_H_20_O	0.02
Dec-2-enal	3913-71-1	C_10_H_18_O	0.04
Deca-2,4-dienal	5910-88-3	C_10_H_16_O	0.04
Undec-2-enal	53 448-07-0	C_11_H_20_O	0.07
Tetradecanal	124-25-4	C_14_H_28_O	0.05
Pentadecanal	2765-11-9	C_15_H_30_O	1.02
Hexadecanal	629-80-1	C_16_H_32_O	0.14
Hexadecenal	76261-03-5	C_16_H_30_O	0.03
6,10,14-Trimethylpentadecan-2-one	16825-16-4	C_18_H_36_O	0.21
Heptadecadienal	56797-42-3	C_17_H_30_O	3.24
Heptadecenal	98028-42-3	C_17_H_32_O	3.58

**Aliphatic alcohols**			
Octan-1-ol	111-87-5	C_8_H_18_O	0.02
Nonan-2-ol	628-99-9	C_9_H_20_O	0.14
Nonan-1-ol	143-08-8	C_9_H_20_O	0.04
Undecan-2-ol	1653-30-1	C_11_H_24_O	0.01
Tridecan-1-ol	61725-89-1	C_18_H_38_O_3_	0.01

**Aromatic compounds**			
2-(*tert*-Butyl)-1,4-dimethoxybenzene	21112-37-8	C_12_H_18_O_2_	0.02

**Fatty acids**			
Dodecanoic acid	143-07-7	C_12_H_24_O_2_	0.07
Tetradecanoic acid	62217-70-3	C_14_H_28_O_2_	0.22
Pentadecanoic acid	1002-84-2	C_15_H_30_O_2_	0.03
Hexadecenoic acid	629-56-1	C_16_H_30_O_2_	0.06
Hexadecanoic acid	57-10-3	C_16_H_32_O_2_	8.34
Octadecenoic acid	2825-79-8	C_18_H_34_O_2_	77.79
Octadecanoic acid	85541-42-0	C_18_H_36_O_2_	0.16
Icosenoic acid	7050-07-9	C_20_H_38_O_2_	0.49

**Esters**			
Methyl tetradecanoate	124-10-7	C_15_H_30_O_2_	0.02
Methyl hexadecanoate	112-39-0	C_17_H_34_O_2_	0.20
Ethyl hexadecanoate	628-97-7	C_18_H_36_O_2_	0.07
Isopropyl hexadecanoate	142-91-6	C_19_H_38_O_2_	0.03
Methyl octadecadienoate	112-63-0	C_19_H_34_O_2_	0.40
Methyl octadecenoate	14620-36-1	C_19_H_36_O_2_	1.06
Ethyl octadecenoate	1260505-83-6	C_20_H_38_O_3_	0.40

**Monoterpenoids**			
Methyl icosenoate	2390-09-2	C_21_H_40_O_2_	0.19
Estragole	140-67-0	C_10_H_12_O	0.06
β-Ionone	79-77-6	C_13_H_20_O	0.02

**Sesquiterpenoids**			
Copaene	138874-68-7	C_15_H_24_	0.01
β-Caryophyllene	87-44-5	C_15_H_24_	0.04
α-Bergamotene	17699-05-7	C_15_H_24_	0.02
β-Farnesene	3899-18-1	C_15_H_26_	0.04
Germacrene D	37839-63-7	C_15_H_24_	0.09
β-Selinene	17066-67-0	C_15_H_24_	0.01
α-Muurolene	10208-80-7	C_15_H_24_	0.02
Himachalene	1461-03-6	C_15_H_24_	0.17
Cadinene	523-47-7	C_15_H_24_	0.18
Carotol	465-28-1	C_15_H_26_O	0.08
Cedrol	77-53-2	C_15_H_26_O	0.09
Dihydrofarnesol	51411-24-6	C_15_H_28_O	0.04

**Higher isoprenoids**			
Squalene	111-02-4	C_30_H_50_	0.17

**Others**			
2-Isopropyl-3-methoxypyrazine	25773-40-4	C_8_H_12_N_2_O	0.03

## 3. Biological activities

The crude extracts and isolated compounds (mainly DAs and flavanols) of *Consolida* plants have been widely screened for their bioactivity. Preliminary screening tests revealed that *Consolida*-derived constituents possessed broad and impressive biological activities, including insecticide, antileishmanial, antimicrobial, antiviral, antitumor, and antioxidant activities. Herein, the biological activities of the crude extracts and isolated compounds of *Consolida* plants are summarized and discussed.

### 3.1. Insecticidal activity

Similar to its related genera *Aconitum* and *Delphinium*, the extracts or powders from plants in the *Consolida* genus have also been used widely as natural insecticides against various kinds of agricultural pests, which indicates that the constituents of *Consolida* plants possess insecticidal activities. Early in the mid-1980s, it was reported that the C_19_-DA methyllycaconitine, which can also be found in *Consolida* plants, displayed high affinity to insect nicotinic receptors and had evolved to protect plants against pests in their early growth stages.^77,78^ Thus, the DAs in *Consolida* may play a vital role in the insecticidal activities of *Consolida* plants, and the results from several studies seem to support this viewpoint. Ulubelen *et al.* tested the insect repellent activities of 29 natural DA components, six of which (79, 80, 84, 99, 112 and 115) were isolated from Turkish *Consolida* species, against a common household pest, the red flour beetle (*Tribolium casteneum* Herbst.).^79^ C_20_-DA hetisine (112) (repellency of 59.12% at 3 mg mL^−1^) was found to have the highest activity among all tested alkaloids, suggesting that it is a promising candidate for insecticide development. In addition, the C_19_-DAs lycoctonine (80) and browniine (84) and the C_20_-DA ajaconine (100) also showed a repellency class III effect (40.1–60%) with repellency values of 46.87%, 46.87%, and 53.12% at 3 mg mL^−1^, respectively, while delsoline (79) and hetisinone (115) showed only a low class II repellent effect (both with a repellency value of 37.50% at 3 mg mL^−1^).

A series of C_19_- and C_20_-DAs isolated from *Consolida* species were evaluated for their insect antifeedant activities on polyphagous *Spodoptera littoralis* and the Colorado potato beetle *Leptinotarsa decemlineata*, as well as their toxicity to insect-derived Sf9 cells (derived from *S. frugiperda* pupal ovarian tissue) and mammalian Chinese hamster ovary (CHO) cells (Table 3).^80,81^ Most of the tested DAs showed notable antifeedant effects on these two pests (EC_50_ < 50 μg cm^−2^), and the antifeedant effects of DAs were found to be species- and structure-dependent (Table 4). Overall, DAs were more effective on *L. decemlineata* than on *S. littoralis*. Among these *Consolida*-derived DAs, the most active antifeedant to *L. decemlineata* was lycaconitine-type C_19_-DA 8-*O*-methylconsolarine (57, EC_50_ = 0.13 μg cm^−2^), followed by lycaconitine-type C_19_-DAs 91, 78, 51, 81, 31, and aconitine-type DA 9 (EC_50_ < 1 μg cm^−2^). Ajadine (31, EC_50_ = 0.1 μg cm^−2^) exerted the strongest antifeedant effect on *S. littoralis*, followed by alkaloids 78 (EC_50_ = 0.84 μg cm^−2^) and 87 (EC_50_ = 1.51 μg cm^−2^). Only a few tested DAs showed toxicity to insect-derived Sf9 cells (LD_50_ < 100 μg mL^−1^), and the most toxic compound was 14-*O*-deacetylpubescenine (58, LD_50_ = 0.38 μg mL^−1^), followed by tuguaconitine (4, LD_50_ = 1.83 μg mL^−1^) and 14-*O*-demethyldelboxine (2, LD_50_ = 6.27 μg mL^−1^). In addition, none of the tested DAs showed cytotoxicity to CHO cells (LD_50_ > 100 μg mL^−1^). In general, C_19_-DAs demonstrated better antifeedant activities than C_20_-DAs, especially lycaconitine-type C_19_-DAs. From the viewpoint of chemical structure, it seemed that lycaconitine-type C_19_-DAs with ester substituents were more effective, but more research is needed for confirmation. The data described above, combined with the fact that more C_19_-DAs are present in *Consolida* plants, indicate that C_19_-DAs play a key role in the insecticidal activity of *Consolida* plants. These results also encourage further in-depth research on the antifeedant activities of *Consolida*-derived C_19_-DAs.

**Table 3:** Antifeedant effects of DAs on *L. decemlineata* and *S. littoralis* and cytotoxicity on Sf9 cells

Compounds	Type	*L. decemlineata* (EC_50_, μg cm^−2^)	*S. littoralis* (EC_50_, μg cm^−2^)	Sf9 cells (LD_50_, μg mL^−1^)
14-*O*-Demethyldelboxine (2)	I	1.92	≈50	6.27
14-Demethyltuguaconitine (3)	I	2.36	5.38	>100
Tuguaconitine (4)	I	3.31	11.79	1.83
Pubescenine (6)	III	12.53	>50	>100
Raveyine (9)	III	0.99	>50	>100
Ajadelphinine (19)	III	4.43	>50	>100
Ajadine (31)	IV	0.84	0.42	>100
14-Deacetylajadine (32)	IV	nt	nt	>100
18-Hydroxy-14-*O*-methylgadesine (49)	IV	0.13	>50	>100
18-Demethylpubescenine (51)	IV	0.60	>50	29.17
1-*O*,19-Didehydrotakaosamine (56)	IV	1.49	14.29	>100
8-*O*-Methylconsolarine (57)	IV	0.23	>10	>100
14-*O*-Deacetylpubescenine (58)	IV	≈50	17.99	0.38
18-*O*-Benzoyl-14-*O*-deacetyl-18-*O*-demethylpubescenine (59)	IV	nt	nt	>100
18-Methoxygadesine (60)	IV	6.36	>50	>100
Consolidine (61)	IV	≈50	9.86	>100
Olivimine (62)	IV	10.92	>50	>100
Olividine (63)	IV	3.62	3.33	29.45
Gigactonine (75)	IV	13.02	9.31	>100
Delcosine (76)	IV	1.11	3.53	32.37
Deltatsine (78)	IV	0.54	0.84	>100
Delsoline (79)	IV	2.22	>50	>100
Lycoctonine (80)	IV	>50	>50	>100
Takaosamine (81)	IV	0.66	5.29	>100
Delphatine (82)	IV	2.97	2.72	>100
Browniine (84)	IV	nt	Nt	>100
14-Acetyldelcosine (87)	IV	>50	1.51	14.88
14-*O*-Acetyldelectinine (91)	IV	0.29	5.63	>100
Dihydroajaconine (96)	V	5.0	>50	>100
Isoatisine (99)	V	3.4	>50	>100
Ajaconine (100)	V	5.1	8.2	>100
Glandulosine (107)	VI	4.0	>50	>100
Hetisine (113)	VI	1.73	≈50	>100
Atropine		7.38	>50	>100
Anabasine		>50	≈60	>100
Eserine		≈60	>50	>100

**Table 4:** *In vitro* activity of flavonoids of *Consolida* plants on promastigotes of *Leishmania* species

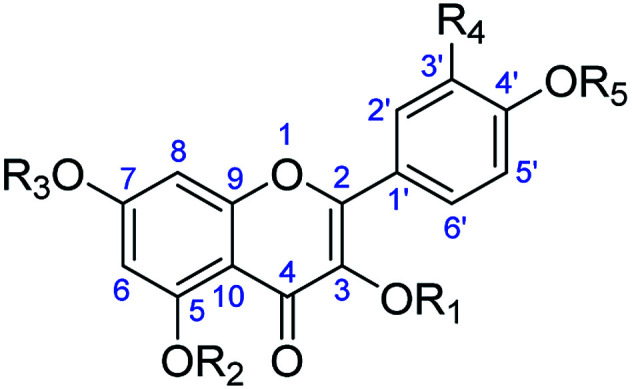			
R_1_–R_5_ (name, no.)	IC_50_ (μM)		
	*L. peruviana*	*L. braziliensis*	J774.2 cells
R_1_ = R_2_ = R_3_ = R_4_ = R_5_ = H (kaempferol,132)	71.29	53.65	53.67
R_1_ = R_2_ = R_3_ = R_4_ = Ac, R_4_ = H (kaempferol tetraacetate, 132a)	53.32	68.56	15.56
R_1_ = R_2_ = R_3_ = R_5_ = H, R_4_ = OH (quercetin, 133)	60.04	30.49	125.44
R_1_ = R_2_ = R_3_ = R_5_ = Ac, R_4_ = OAc (quercetin pentaacetate, 133a)	11.18	46.78	109.23
R_1_ = β-d-Gal, R_2_ = R_3_ = R_4_ = R_5_ = H (trifolin, 134)	53.34	52.46	161.32
R_1_ = β-d-Gal Ac, R_2_ = R_3_ = R_5_ = H, R_4_ = H (trifolin heptaacetate, 134a)	10.53	8.72	148.71
R_1_ = 2-*O*-acetyl-β-d-Gal, R_2_ = R_3_ = R_5_ = H, R_4_ = OH (2′′-acetylhyperoside, 136)	7.35	6.21	122.31
R_1_ = 6-*O*-acetyl-β-d-Gal, R_2_ = R_3_ = R_5_ = H, R_4_ = OH (6′′-acetylhyperoside, 137)	86.95	51.60	61.32
Pentostam	11.32	9.56	12.44
Glucatim	15.33	25.61	15.20

### 3.2. Antiparasitic activity

In some countries, such as Turkey and China, *Consolida* plants have been employed as anthelmintic herbals in traditional medicines.^6,82^ Several studies regarding the antiparasitic effect of the crude extracts and isolated compounds of *Consolida* species support the utilization of *Consolida* plants as anthelmintic herbals. Moreover, these results reveal the high potential of *Consolida*-derived compounds in the treatment of protozoal infections.

Carole *et al.* investigated the antileishmanial activities of 27 plants from Lebanese.^83^ The screened plants were extracted with water, methanol, and dichloromethane. The methanol extracts of *C. rigida* (white larkspur) exhibited significant antiamastigote effects on the intracellular form of *Leishmania* species (IC_50_ = 8.1 μg mL^−1^). Furthermore, the methanol extracts also showed no toxicity to the host cells (THP1 human monocytes, IC_50_ > 250 μg mL^−1^), exhibiting a selectivity index (SI) larger than 30. Notably, of the screened plants, the antileishmanial effects of the methanol extracts of *C. rigida* were next only to the aqueous extracts of *Onosma aucheriana* (IC_50_ = 5.1 μg mL^−1^, SI > 49) and the methanol extracts of *Cytisus syriacus* (IC_50_ = 5.8 μg mL^−1^, SI > 43).

From a total of 64 DAs (41 C_19_-DAs and 23 C_20_-DAs) screened by González *et al.*, only three atisine-type C_20_-DAs displayed antiparasitic effects against *Leishmania infantum* and *Trypanosoma cruzi*, while none of the C_19_-DAs affected the parasites.^80,84,85^ Among these three DAs, azitine (93) has been found in *Consolida* species. Azitine (92) showed promising antileishmanial and antitrypanocidal properties. It was effective *in vitro* both against the extracellular and intracellular forms of *L. infantum* and could not only lower the *in vitro* growth rate of *L. infantum* but also affect the capacity to infect cells and reduce the multiplication of amastigotes. In the *in vitro* experiment, azitine (92) exerted an inhibitory effect against *L. infantum* parasites (IC_50_ = 10.12 μg mL^−1^ after 72 h of culture), which was lower than those obtained by the reference drug pentostam (IC_50_ = 11.32 μg mL^−1^ after 72 h of culture), and exhibited an inhibiting effect against *T. cruzi* epimastigotes (IC_50_ = 67.74 μg mL^−1^ after 72 h of culture). In the intracellular experiment, azitine (92) clearly inhibited the infection rate (approximately 53%) of *L. infantum* in J774A.1 macrophage cells after 48 h of culture. Moreover, this alkaloid is not toxic to host cells (IC_50_ > 200 μg mL^−1^), which highlights its potential as a lead compound in the discovery of drugs for protozoal infections.

Additionally, a set of flavonol glycosides obtained from *C. oliveriana* and their acetylated products have exhibited impressive antileishmaniasis activity against two *Leishmania* species *L. peruviana* and *L. braziliensis* (Table 4).^86–88^ All the compounds tested showed high inhibitory effects against their corresponding parasites, and some of them had higher effectiveness and selectivity indexes than those of their corresponding reference drugs. For example, acetylated compounds 133a, 134a, and 136 were highly active against *L. peruviana*, and 133a and 136 were strongly effective against *L. braziliensis*. Transmission electronic microscopy and nuclear magnetic resonance analysis raised the possibility that the action (or part of the action) could be at the level of the parasite membranes. Regarding structures, the acetylated compounds performed better than the phenolic analogs, and the kaempferol derivatives possessing a monosubstituted B-ring were more active than the quercetin analogs. The interesting structure–activity relationship (SAR) described above implies that the *Consolida*-derived flavonols can serve as a low-cost starting material for the discovery of acetylated compounds with better antileishmaniasis efficacy.

### 3.3. Antimicrobial activity

The crude extracts of several *Consolida* plants have been evaluated for their antimicrobial activities against some kinds of plant and human pathogenic bacteria and fungi. In a screening of plants with antimicrobial activity from northeastern Iran, two *Consolida* species, *C. orientalis* and *C. rugulosa*, were evaluated for their antimicrobial activity against several pathogenic bacteria and fungi, and *C. orientalis* showed significant antimicrobial activity against *Morganella morganii* and *P. aeruginosa*.^89^ Ucar tested the antimicrobial activity of ethanol extracts from the aerial parts (leaf, flower, and branch) of *C. regalis* against a series of common human pathogenic bacteria and fungi, including *Staphylococcus aureus*, *Enterococcus faecalis*, *Pseudomonas aeruginosa*, *Escherichia coli*, *Klebsiella pneumonia*, *Bacillus cereus, Candida albicans*, and *C. tropicalis*. The extracts from the leaf, flower, and branch parts of *C. regalis* showed moderate antimicrobial activity against *C. tropicalis* with MIC values of 0.625 mg mL^−1^, 0.625 mg mL^−1^, and 0.312 mg mL^−1^, respectively, and the extracts from the leaf and branch parts showed moderate antimicrobial activity against *S. aureus*, with MIC values of 0.625 mg mL^−1^. The extracts showed only a weak effect on the other tested microorganisms.^90^

Kalpana *et al.* evaluated the antifungal activities of methanol extracts from the leaves, stems and flowers of *C. ambigua* (*D. ajacis*) against several phytopathogenic fungi, *Alternaria solani*, *Rhizoctonia solani*, *Colletotrichum gloeosporioides* and *Pyricularia oryzae*. All of these extracts at 10 mg mL^−1^ were effective at inhibiting fungal colony growth compared with that of the control. The extract of the *C. ambigua* leaves showed the complete inhibition of *P. oryzae* colony growth, followed by the almost complete inhibition of *C. gloeosporioides* colony growth, whereas low inhibition was observed against *R. solani* and *A. solani*. The stem extract showed the complete inhibition of the colony growth of *C. gloeosporioides*, *P. oryzae* and *R. solani*, followed by the inhibition of *A. solani* colony growth; the flower extract completely reduced the growth of the plant pathogenic fungus *C. gloeosporioides*, followed by *P. oryzae* while the least inhibition was observed against *A. solani*.^91^ In addition, Yusuf *et al.* tested the antifungal activity of the leaf extracts of *D. consolida* against *Alternaria solani*, an early blight disease pathogen of potato. However, the studied extracts showed no inhibitory effect on the mycelial growth of *A. solani*.^92^

The above antimicrobial activities can be attributed to their DA compositions, which have been reported to exhibit certain antibacterial and antifungal activities.^15^ Bilge *et al.* reported that five *Consolida* alkaloids presented a notable antibacterial effect only toward *K. pneumoniae* and *A. baumannii* with MIC values of 8 μg mL^−1^, while the five *Consolida* alkaloids exhibited considerable antifungal activity with MIC values of 4 μg mL^−1^ (Table 5).^93^

**Table 5:** Antimicrobial activities of DAs

DAs	*E. coli*	*P. aeruginosa*	*P. mirabilis*	*K. pneumoniae*	*A. baumannii*	*S. aureus*	*B. subtilis*	*C. albicans*
Lycoctonine (80)	32	64	32	8	8	64	128	4
18-*O*-Methyllycoctonine (61)	32	64	32	8	8	64	128	4
Delcosine (76)	32	64	32	8	8	64	128	4
14-Acetyldelcosine (87)	32	64	32	8	8	64	128	4
14-Acetylbrowniine (84)	32	64	32	8	8	64	128	4
Ampicilline	2	—	2	2	2	<0.12	0.12	—
Oflaxocine	0.12	1	<0.12	0.12	0.12	0.5	0.5	—
Ketocanazole	—	—	—	—	—	—	—	2

### 3.4. Antiviral activity

The isolated DAs of *Consolida* plants, mainly lycaconitine-type C_19_-DAs, show antiviral activities toward several highly pathogenic viruses. Five known lycaconitine-type DAs from Turkish *Consolida* species were screened for their antiviral effects on both DNA virus herpes simplex (HSV) and RNA virus parainfluenza (PI-3) using Madin–Darby bovine kidney and Vero cell lines. The maximum non-toxic concentrations (MNTC) and cytopathogenic effects (CPE) were determined using acyclovir and oseltamivir as the references. Consequently, a selective inhibition was observed toward PI-3 virus by these alkaloids, while they were entirely unsuccessful in the inhibition of HSV (Table 6). The PI-3 inhibitory activity of the alkaloids was fairly analogous to that of the positive control oseltamivir, ranging between 8–32 μg mL^−1^ as the minimum and maximum inhibitory concentrations for the cytopathogenic effect (CPE).^93^ In addition, the new lycaconitine-type C_19_-DAs ajacisines C–E (37–39) and isodelectine (47) were found to exhibit moderate to weak antiviral effects against respiratory syncytial virus (RSV) with IC_50_ values of 75.2, 35.1, 10.1, and 50.2 μM, respectively,^41^ while the positive control ribavirin showed an IC_50_ value of 3.1 μM. The antiviral activities of DAs may be due to their high reactivity with microtubules, thus destroying their stability by polarity; this result can block cellular division and prevent the rapid growth of cancer cells.^94^

**Table 6:** Antiviral effects of DAs against HSV and PI-3

Compounds	MDBK cells (MNTC, μg mL^−1^)	HSV		Vero cells (MNTC, μg mL^−1^)	PI-3	
		Max.	Min.		Max.	Min.
Lycoctonine (80)	64	—	—	32	32	8
18-*O*-Methyllycoctonine (61)	64	—	—	64	32	1
Delcosine (76)	64	—	—	64	32	1
14-Acetyldelcosine (87)	64	—	—	64	32	1
14-Acetylbrowniine (84)	64	—	—	64	32	1
Acyclovir	16	16	<0.25	—	—	—
Oseltamivir	—	—	—	32	32	<0.25

### 3.5. Antitumor activity

Although no species of *Consolida* are traditionally used to treat cancer, several studies have revealed that the crude extracts and isolated compounds of *Consolida* plants possess certain antitumor effects. In a screening of anticancer plants from Iran, the ethanol extracts of *C. orientalis* exerted an antiproliferative effect against human cervical carcinoma HeLa cells with an IC_50_ value of 1.6 mg mL^−1^,^95^ which might be attributed to the high content of some DAs with cytotoxic activities in the *C. orientalis* extracts.^17,96^

De Inés *et al.* evaluated the cytotoxic effects of 43 DAs (40 C_19_-DAs and 3 C_18_-DAs) on CHO cells and several tumor cell lines, including CT26 (murine colon adenocarcinoma), SW480 (human colon adenocarcinoma), HeLa, SkMel25 (human melanoma) and SkMel28 (human malignant melanoma).^97^ As shown in Table 7, 13 of the tested alkaloids that have been found in *Consolida* plants produced a cytotoxic effect on the different cell lines (MICs < 100 μg mL^−1^). Among the various groups, the most active alkaloids were found among the lycaconitine-type C_19_-DAs. All the cell lines responded to 27, 56 and 60 with varying potencies. Alkaloid 27 was the most cytotoxic to CHO and SkMel28, while 56 was the most cytotoxic to CT26, SW480, HeLa and SkMel25 cells, indicating selective structure-dependent cytotoxicity for the group. Alkaloids 13 and 19 also showed relatively strong cytotoxicity toward several tumor cell lines. It is worth noting that most of the active alkaloids, including the most effective alkaloid 56, exhibited selective cytotoxicity to cancerous *versus* noncancerous tissues, which highlights their potential use as candidates for the treatment of cancer. In addition, the viability assays indicated that their cytotoxic effects could be related to the inhibition of ATP production.

**Table 7:** Antitumor effects of DAs against human cancer cell lines

Compounds	MICs (μg mL^−1^)					
	CHO	CT26	SW480	Hela	SkMel25	Skmel28
Pubescenine (6)	>100	100	25	50	50	>100
Raveyine (9)	>100	50	50	>100	50	>100
Neoline (13)	>100	25	12.5	6.25	25	>100
Ajadelphinine (19)	>100	50	25	12.5	25	>100
Didehydrodelsoline (27)	6.25	12.5	12.5	12.5	25	6.25
Ajadine (31)	50	50	50	>100	>100	50
14-Deacetylajadine (32)	>100	>100	100	50	100	>100
Methyllycaconitine (48)	12.5	12.5	50	50	100	100
18-Demethylpubescenine (51)	>100	>100	>100	>100	50	>100
1-*O*,19-Didehydrotakaosamine (56)	>100	6.25	6.25	0.4	6.25	25
18-Methoxygadesine (60)	25	50	25	25	25	>100
Lycoctonine (80)	>100	50	50	>100	>100	>100
Delphatine (82)	>100	>100	>100	100	>100	>100

While flavanol glycosides from *Consolida* themselves are slightly active against certain human cancer cell lines, increasing cytotoxic activity has been observed after the corresponding flavanols undergo acetylation. Diaz *et al.* prepared a series of flavanol acetates isolated from the aerial parts of *C. oliveriana* and tested their cytotoxicity effects against the human myeloid leukemia HL-60 and U937 cell lines and the human melanoma SK-MEL-1 cell line (Table 8).^59^ As shown in Table 8, some of these flavonol glycoside acetates (132a, 133a, 134a, 134b and 135a) displayed cytotoxicity against the tested cancer cell lines with IC_50_ values ranging from 10 to 88 μM. In particular, trifolin heptaacetate (134a) was the most effective against all assayed cell lines, with an IC_50_ value of approximately 10–15 μM. A subsequent pharmacological study revealed that trifolin heptaacetate could induce cancer cell apoptosis through a caspase-dependent mechanism that is associated with the release of cytochrome c.^98^ It has been suggested that trifolin heptaacetate has the potential to be developed as a chemopreventive agent and possibly as a therapeutic agent against cancer; however, more detailed mechanistic studies on trifolin heptaacetate are still needed.

**Table 8:** Antitumor effects of flavanol glycosides against human cancer cell lines

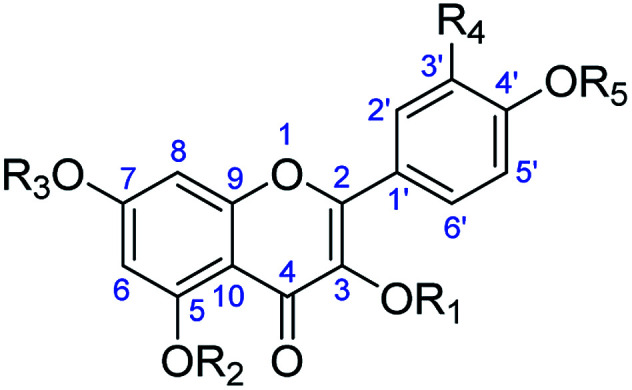			
R_1_–R_5_ (name, no.)	IC_50_ (μM)		
	HL-60	U937	SK-MEL-1
R_1_ = R_2_ = R_3_ = R_4_ = Ac, R_4_ = H (kaempferol tetraacetate, 132a)	45	48	37
R_1_ = R_2_ = R_3_ = R_5_ = Ac, R_4_ = OAc (quercetin pentaacetate, 133a)	38	25	58
R_1_ = β-d-Gal Ac, R_2_ = R_3_ = R_5_ = H, R_4_ = H (trifolin heptaacetate, 134a)	21	10	15
R_1_ = β-d-Gal OMe, R_2_ = R_3_ = R_5_ = Me, R_4_ = H (heptamethyltrifolin, 134b)	88	>100	>100
R_1_ = β-d-Gal Ac, R_2_ = R_3_ = R_5_ = Ac, R_4_ = OAc (hyperoside acetate, 135a)	15	19	23
R_1_ = 2-*O*-acetyl-β-d-Gal, R_2_ = R_3_ = R_5_ = H, R_4_ = OH (2′′-acetylhyperoside, 136)	>100	>100	>100
R_1_ = 6-*O*-acetyl-β-d-Gal, R_2_ = R_3_ = R_5_ = H, R_4_ = OH (6′′-acetylhyperoside, 137)	>100	>100	>100
R_1_ = β-d-Gal Ac, R_3_ = β-d-Glu Ac, R_2_ = R_5_ = Ac, R_4_ = H (glucotrifolin acetate, 138a)	>100	>100	>100

### 3.6. Antioxidant activity

Several *Consolida* species have been evaluated for their antioxidant activities by using *in vitro* antioxidant assays, and considerably different effects have been observed. Zeng *et al.* reported that the aqueous extracts of *C. ambigua* (*D. ajacis*) flowers exhibited only a weak DPPH radical scavenging ability among 45 tested flowers, although these *C. ambigua* extracts could effectively scavenge hydroxyl superoxide and anion radicals.^99^ In contrast, investigations performed by Zengin *et al.* and Zengin *et al.* showed that *C. orientalis* has powerful antioxidant activities, effectively scavenging free radicals, including DPPH, ABTS, and superoxide radicals; reducing cupric and ferric ions; chelating prooxidant metal ions; and inhibiting the oxidation of linoleic acid.^100,101^ Another investigation also demonstrated that *C. regalis* possesses a powerful ability to scavenge DPPH and ABTS free radicals.^90^ The difference in antioxidant activities between these *Consolida* species may be attributed to their different phenolic contents.

## 4. Conclusions

To the best of our knowledge, a total of 143 distinct compounds, including 126 alkaloids (121 DAs and 5 other alkaloids) and 17 flavonoids (5 anthocyanins and 12 flavanols), have been isolated and identified from *Consolida* plants, which indicate that the *Consolida* genus is a source of abundant DAs. The DAs that have been found in *Consolida* plants consist of 5 C_18_-DAs, 87 C_19_-DAs and 29 C_20_-DAs. In terms of DA subtypes, the lycaconitine-type C_19_-DAs with 73 members account for the largest proportion (58%) of the isolated alkaloids; thus, lycaconitine-type C_19_-DAs can be regarded as the characteristic and representative components of the genus *Consolida*. On the other hand, of the 143 isolated compounds, 73 are novel, including 69 new DAs and 4 new anthocyanins. Among these new compounds, several possess unprecedented structures or uncommon substituents. These findings underscore the high chemical diversity among the chemical constituents of *Consolida* plants, which can serve as a vast resource for drug discovery.

The crude extracts and isolated compounds of *Consolida* plants have been reported for their various biological activities, including insecticidal, antiparasitic, antifungal, antiviral, anticancer, and antioxidant activities. Some of the reported effects are in accordance with the purported uses of *Consolida* plants in folk medicine, which is conducive to illuminating the pharmacodynamic material basis of *Consolida*-derived herbal drugs. For example, the anthelmintic effects of *Consolida* plants may be attributed to the anthelmintic effects of DAs. Some constituents from *Consolida* plants possess activities that differ from their traditional medicinal use, such as antitumor and antioxidant activities, indicating the novel potential applications for the use of *Consolida* plants.

Although phytochemical and biological studies on *Consolida* plants have attracted considerable interest, some research potential remains. First, of the 50 *Consolida* species around the world, only a few species have been studied for their biological constituents. The related investigations are restricted to the widespread *Consolida* species, such as *C. ambigua*, which contributes relatively more compounds than other species. Most of the less common *Consolida* species are still largely unstudied. Hence, an extensive investigation of the other *Consolida* species, especially species that are used medicinally, remains necessary.

Second, the preliminary detection performed by using LC, GC, and MS techniques reveal that there are a number of other compounds in *Consolida* plants, such as phenolic acids, steroids, FAs and volatile constituents, that may also possess new structures or notable biological activities, thus potentially serving as a medicinal resource for drug discovery. In addition, unlike toxic DAs, the phenolic acids, steroids, FAs and volatile constituents are generally less toxic, which is advantageous for the food and pharmaceutical industry. However, these compounds have not attracted the interest of researchers, and none have been isolated. Thus, further studies on the isolation and biological tests of these compounds are strongly encouraged.

Finally, all of the biological activities of *Consolida* plants have been investigated by using *in vitro* chemical and cellular models, and little clinical or *in vivo* research is currently available. These pharmacological studies are insufficient to validate the effects of *Consolida* plants and their derived compounds, which hinder their application and promotion. It is necessary to evaluate the biological activities of the constituents from *Consolida* plants using both *in vitro* and *in vivo* pharmacological models to facilitate further research and exploitation of this genus.

## Conflicts of interest

There are no conflicts to declare.

## Supplementary Material
